# COVID-19 lockdown highlights impact of recreational activities on the behaviour of coral reef fishes

**DOI:** 10.1098/rsos.220047

**Published:** 2022-11-09

**Authors:** William E. Feeney, Zara-Louise Cowan, Frédéric Bertucci, Rohan M. Brooker, Gilles Siu, Frédérique Jossinet, Tamatoa Bambridge, René Galzin, David Lecchini

**Affiliations:** ^1^ Department of Biosciences, Durham University, Durham DH1 3LE, UK; ^2^ Department of Behavioural Ecology and Evolutionary Genetics, Max Planck Institute of Ornithology, Seewiesen, Germany; ^3^ Centre for Planetary Health and Food Security, Griffith University, Nathan 4111, Australia; ^4^ Ministry of Education Key Laboratory for Ecology of Tropical Islands, Key Laboratory of Tropical Animal and Plant Ecology of Hainan Province, College of Life Sciences, Hainan Normal University, Haikou 571158, People's Republic of China; ^5^ Department of Zoology, The David Attenborough Building, University of Cambridge, Cambridge CB2 3QZ, UK; ^6^ PSL Université Paris, EPHE-UPVD-CNRS, UAR3278 CRIOBE, 98729 Moorea, French Polynesia; ^7^ Functional and Evolutionary Morphology Lab, University of Liège, 4000 Liège, Belgium; ^8^ Centre for Integrative Ecology, School of Life and Environmental Sciences, Deakin University, Queenscliff 3225, Australia; ^9^ Laboratoire d'Excellence ‘CORAIL’, 66100 Perpignan, France

**Keywords:** coral reefs, COVID-19, ecology

## Abstract

In 2020, the COVID-19 pandemic led to a reduction in human activities and restriction of all but essential movement for much of the world's population. A large, but temporary, increase in air and water quality followed, and there have been several reports of animal populations moving into new areas. Extending on long-term monitoring efforts, we examined how coral reef fish populations were affected by the government-mandated lockdown across a series of Marine Protected Area (MPA) and non-Marine Protected Area (nMPA) sites around Moorea, French Polynesia. During the first six-week lockdown that Moorea experienced between March and May 2020, increases (approx. two-fold) in both harvested and non-harvested fishes were observed across the MPA and nMPA inner barrier reef sites, while no differences were observed across the outer barrier sites. Interviews with local amateur and professional fishers indicated that while rules regarding MPA boundaries were generally followed, some subsistence fishing continued in spite of the lockdown, including within MPAs. As most recreational activities occur along the inner reef, our data suggest that the lockdown-induced reduction in recreational activities resulted in the recolonization of these areas by fishes, highlighting how fish behaviour and space use can rapidly change in our absence.

## 1. Introduction

The onset of the ongoing SARS-CoV-2 (COVID-19) pandemic led to the unprecedented closing of international borders, halting of commercial activities and lockdown of resident populations [1]. These sudden changes have recalibrated the impacts that humans are having on the world's ecosystems, with reports suggesting increases in air and water quality [2], the reduction of various pollutants [3,4] and the encroachment of species into areas left absent by humans [5,6]. While the impacts of lockdown may offer initial relief to ecosystems, the resulting breakdown of supply chains and reduction in employment may also present challenges, especially to remote populations, leading to a greater reliance on harvesting resources directly from the surrounding environment [7]. Under these circumstances, this ‘anthropause’ presents a unique opportunity to investigate the impacts that human activities have on animal populations [8].

Over the past half century, the remote island nations of the Pacific have transitioned from relying on subsistence agriculture and fishing to emerging economies that rely primarily on tourism [9]. For instance, the tourism-dominated services sector of French Polynesia accounted for 85% of total value added to its economy in 2012, with 17% of the workforce being employed within the tourism industry [9]. Despite this ongoing transformation, the populations of these countries still rely heavily on local fisheries, with fish comprising the primary source of protein throughout the region and most families having at least one member who fishes in either an amateur or professional capacity to help feed their family [10,11]. Following the identification of the first COVID-19 infection in French Polynesia on 10 March 2020 and the World Health Organization declaring COVID-19 a pandemic on 11 March 2020, the country enforced a total lockdown from 20 March 2020 to 4 May 2020 (six weeks) [12,13]. In the week preceding the lockdown approximately 3000 tourists were repatriated from French Polynesia, and during the lockdown all international travel was suspended with inter-island travel within French Polynesia only possible on presentation of an exemption [12]. Thus, during the lockdown period, French Polynesia became isolated from the rest of the world, with all tourism-based marine activities stopped and only professional fishers being allowed to go to sea. Lockdown then eased across two steps: (1) from 5 May to 30 June 2020, there were no international flights and all hotels remained closed, but residents were allowed to travel between islands; and (2) from 1 July 2020, international flights began to resume and hotels started opening to local and international tourism [12].

**Figure 1.**
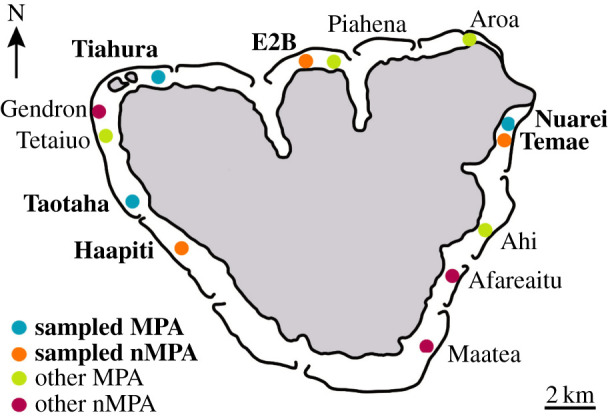
Map of sampling sites around Moorea, French Polynesia.

In 2004, the Fisheries Service of French Polynesia and the Centre de Recherches Insulaires et Observatoire de l'Environnement (CRIOBE) set up a monitoring plan allowing a statistically rigorous assessment of the biological effects of implementing the Marine Protected Areas at Moorea [14]. Thus, each February, eight Marine Protected Area (MPA) and five non-Marine Protected Area (nMPA) sites are surveyed along fixed transects around the island (figure 1). In response to the pandemic and associated lockdown/reduction in normal human activities across the reefs (20 March 2020–4 May 2020), CRIOBE's resident scientists conducted surveys across a limited number of sites. First, in May (5–6), immediately (<48 h) following the lockdown period ending, surveys were conducted to obtain a snapshot of the environment following six weeks of lockdown (‘lockdown’ surveys). These were then repeated in July (6–7) after the tourism industry began re-opening (‘post-lockdown’ surveys). Similar to recent studies on coral reefs in French Polynesia [15], India [16] and Israel [6], we hypothesized that the lockdown-associated reduction in human activities would lead to increases in fish densities across heavily used sites at Moorea. Here, we build on a long-term monitoring effort that uses the aforementioned transects to document fish density and examine how the abrupt reduction of marine-based activities during lockdown affected fish populations within and outside of MPAs at Moorea. Furthermore, as some evidence suggests that poaching rates may have increased during lockdown periods [5], and given the widespread reliance on tourism for much of the nation's employment [9], we also deployed social surveys to local amateur and professional fishers to gain insights into the impacts this had on their fishing practices.

## 2. Material and methods

### 2.1. Fish surveys across a Marine Protected Area network around Moorea

Each February (2004–2020), CRIOBE conducted surveys along transects at three permanent monitoring sites within three MPAs and three nMPAs around Moorea [14,17]. Surveyed areas extended from the shore to the outer reef slope, with each permanent monitoring site within an MPA/nMPA falling within one of three distinct habitat types: the shallow reef flat (or fringing reef), the barrier reef, and the outer slope (at 12 m depth) (figure 1). As a result of the COVID-19 pandemic, CRIOBE experienced reduced research capacity, as many researchers returned to their home countries prior to the onset of the lockdown. Given this, only the barrier and outer slope sites were used to investigate the effect of the lockdown on fish density, which included one MPA and nMPA site on the west coast, one MPA and nMPA site on the north coast, and one MPA and nMPA site on the east coast of Moorea (figure 1). The barrier and outer slope sites were prioritized as they host the majority of adult fish [18,19]. Consequently, no fringing reef sites were included in this study.

Three 25 m transects were conducted at each permanent monitoring site between 08.00 and 11.00, during which all fish within 2 m of the transect tape were recorded. Two passes were conducted during each transect: mobile fish were recorded during the first pass as they can quickly leave the survey area, and more cryptic fishes were the focus of the second pass [17]. During each transect, all adult fishes were identified to species, which were then classified as either harvested by fishers or not harvested by fishers [20]. ‘Lockdown’ transects were conducted on the 5–6 May, within 48 h of lockdown being lifted, and ‘post-lockdown’ transects in July (6–7), approximately two months following lockdown being lifted, when international tourists were present again at Moorea. Transects at a given location were conducted with a 25 m gap between each replicate.

### 2.2. Fisher attitudes towards the COVID-19 lockdown

Following lockdown, local fishers that lived in front of MPAs and nMPAs around Moorea were interviewed using a standardized set of binary-choice questions. Participants were interviewed anonymously about the health and economic crisis that COVID-19 presented, as well as whether eating habits, use of local fisheries, and respect for no-take rules within MPAs had been affected by the lockdown. The interviews lasted less than 10 min and answers were recorded using a pencil and paper. Only residents of Moorea (maximum one member per household) that self-identified as professional or amateur fishers were interviewed. All interviewees (six professional fishers and 63 amateur fishers) were asked to answer the following questions: Were you afraid to catch COVID-19 during the lockdown? Were you financially impacted by the crisis? Did you change your diet during the lockdown? Did you eat more locally harvested fruits and vegetables, or fishes? In addition to these questions, the six professional fishers were also asked: Did you continue to fish as usual during the lockdown period? If not, why? The 63 amateur fishers were also asked: Did you respect the rules of lockdown with regards to the fishing rules and regulations? If not, why, when and where did you fish? Interviews were conducted in French, with exception of fish names, which were discussed in Tahitian.

### 2.3. Statistical analysis

To test if the densities of harvested and non-harvested fish species were affected by the COVID-19 lockdown, we investigated whether survey data from lockdown and post-lockdown transects differed within MPA and nMPA barrier reef and outer slope sites compared to the 10-year average (February 2011–February 2020). Data from previous years were not included as Moorea experienced a crown-of-thorns starfish (*Acanthaster* cf. *solaris*) outbreak between 2006 and 2009, and a cyclone in 2010, which comprise disturbances not relevant to this study [17,21]. Data analyses were performed in R, v. 4.0.0 [22] using RStudio, v. 1.4.1106 [23], and linear-mixed effects models were conducted using the {lme4} package [24]. All full and final models included the following variables: fish density as the dependent variable; time period (long-term average [February 2011–2020], lockdown [May 2020] and post-lockdown [July 2020]) as the fixed effect; and site (Nuarei, Tiahura and Taotaha [MPA sites], or Temae, Entre 2 Baies and Haapiti [nMPA sites]) as a random effect with transect (T1–3) nested within site. Model assumptions for each model were examined using the {DHARMa} package [25]. When the residual diagnostics indicated a significant deviation from the expected distribution (i.e. significant Kolmogorov–Smirnov test) we log10 transformed the dependent variable. This was conducted for three models: barrier reef, MPA, non-harvested; outer slope, MPA, harvested; and outer slope, nMPA, harvested. When there was a significant difference in fish density across time periods, *post hoc* Tukey tests were conducted using the {emmeans} package [26]. Data were plotted using the {ggplot2} [27] package. An R Markdown document containing all analyses is provided in the electronic supplementary material.

## 3. Results

### 3.1. Fish surveys across a Marine Protected Area network around Moorea

**Figure 2.**
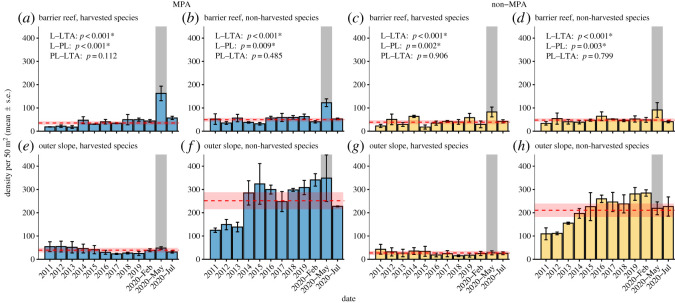
Density (mean ± s.e.) per 50 m^2^ of harvested (*a,c,e,g*) and non-harvested (*b,d,f,h*) fish between 2011 and 2020 at six locations (three MPA (*a,b,e,f*) and three non-MPA (*c,d,g,h*)), measured on both the barrier reef (*a–d*) and outer slope (*e–h*). Surveys were conducted during February between 2011 and 2020, with additional surveys conducted in May 2020 (immediately following the six-week COVID-19 lockdown, i.e. ‘lockdown’ survey; highlighted by a shaded bar) and July 2020 (two months following the removal of COVID-19 lockdown, i.e. ‘post-lockdown’ survey). Dashed red line indicates the 10-year ‘long-term’ average with 95% CI (calculated from density measurements from 2011 to February 2020). Results of *post hoc* tests are included where ANOVA/Kruskal–Wallis tests indicated there was a significant difference in mean fish density between the three groups (L = lockdown, PL = post-lockdown, LTA = long-term average); asterisk indicates a significant difference between two groups.

There was a significant difference in the density of both harvested (χ_2_^2^ = 91.071, *p* < 0.001, figure 2*a*), and non-harvested ($\chi_{2}^{2}=25.055$, *p* < 0.001, figure 2*b*) fishes between the three time periods (lockdown, post-lockdown and 10-year ‘long-term’ average) at the barrier reef MPA sites. *Post hoc* tests indicated that fish density recorded during the lockdown period was significantly greater than the long-term average and the post-lockdown surveys for both harvested (both *p* < 0.001) and non-harvested (*p* < 0.001 and *p* = 0.009, respectively) species, while there was no difference between post-lockdown and the long-term average for both the harvested (*p* = 0.112) and non-harvested (*p* = 0.485) species (figure 2*e*,*f*). We also found a significant difference in the density of harvested ($\chi_{2}^{2}=23.783$, *p* < 0.001; figure 2*c*) and non-harvested ($\chi_{2}^{2}=15.638$, *p* < 0.001; figure 2*d*) species at the nMPA barrier reef sites. Again, *post hoc* tests indicated that fish density recorded during the lockdown period was significantly greater than the long-term average and the post-lockdown surveys for both harvested (*p* < 0.001 and *p* = 0.002, respectively) and non-harvested species (*p* < 0.001 and *p* = 0.003, respectively), while there was no significant difference between post-lockdown and the long-term average for both the harvested (*p* = 0.906) and non-harvested (*p* = 0.799) species (figure 2*c*,*d*, respectively). For the outer slope sites, there was no significant difference in fish density across the three time periods at the MPA sites for either harvested ($\chi_{2}^{2}=3.036$, *p* = 0.219; figure 2*e*) or non-harvested ($\chi_{2}^{2}=4.543$, *p* = 0.103; figure 2*f*) species, nor at the nMPA sites for either harvested ($\chi_{2}^{2}=2.625$, *p* = 0.269, figure 2*g*) or non-harvested ($\chi_{2}^{2}=0.313$, *p* = 0.855, figure 2*h*) species.

Within the MPA barrier reef sites, a taxonomically and functionally diverse range of species recorded an increase in density during the lockdown surveys compared to the long-term average. For example, the five harvested species that experienced the greatest increase included: convict surgeonfish (manini [Tahitian name]; *Acanthurus triostegus*) (0.41 per 50 m^2^ long-term average compared to 25.56 during lockdown); honeycomb grouper (tārao; *Epinephelus merra*) (0.11 per 50 m^2^ long-term average compared to 3.78 during lockdown); striped large-eye bream (māene; *Gnathodentex aureolineatus*) (0.16 per 50 m^2^ long-term average compared to 5.11 during lockdown); blacktail snapper (to'au; *Lutjanus fulvus*) (0.14 per 50 m^2^ long-term average compared to 2.89 during lockdown); and sabre squirrelfish (apa'i; *Sargocentron spiniferum*) (0.03 per 50 m^2^ long-term average compared to 0.67 during lockdown) (electronic supplementary material, table S1). Four species were also recorded for the first time during the lockdown (whitespotted surgeonfish [api; *Acanthurus guttatus*] [0.11 recorded during lockdown], scarlet soldierfish [ī'ihi; *Myripristis pralinia*] [8.33 per 50 m^2^ recorded during lockdown], orangespine unicornfish [ume tārei; *Naso lituratus*] [1.33 per 50 m^2^ recorded during lockdown], and Tahitian squirrelfish [māuna'una; *Sargocentron tiere*] [0.33 per 50 m^2^ recorded during lockdown]) (electronic supplementary material, table S1).

Similar patterns were also found for the non-harvested species at the MPA barrier reef sites. Notable increases were recorded in: scissortail sergeant (mamo; *Abudefduf sexfasciatus*) (0.44 per 50 m^2^ long-term average compared to 3.33 during lockdown), blackspot sergeant (pa'e'e; *Abudefduf sordidus*) (0.1 per 50 m^2^ long-term average compared to 6.89 during lockdown), bluntheaded wrasse (po'au; *Thalassoma amblycephalum*) (0.06 per 50 m^2^ long-term average compared to 2.56 during lockdown), arc-eye hawkfish (pātu'i; *Paracirrhites arcatus*) (0.08 per 50 m^2^ long-term average compared to 2.22 during lockdown) and sixstripe wrasse (po'au, *Pseudocheilinus hexataenia*) (0.13 per 50 m^2^ long-term average compared to 3.33 during lockdown) (electronic supplementary material, table S2). Again, four species were also recorded for the first time during the lockdown (white-spotted puffer [huehue; *Arothron hispidus*] [0.11 per 50 m^2^ recorded during lockdown], melon butterflyfish [pāraharaha; *Chaetodon trifasciatus*] [2.67 per 50 m^2^ recorded during lockdown], whitespotted boxfish [moemoe; *Ostracion meleagris*] [0.33 per 50 m^2^ recorded during lockdown] and surge wrasse [po'au a'au; *Thalassoma purpureum*] [0.11 per 50 m^2^ recorded during lockdown]) (electronic supplementary material, table S2).

A taxonomically and functionally diverse range of fish species also experienced an increase in density within the nMPA barrier reef sites during the lockdown surveys compared to the long-term average. The five harvested species that showed the largest increases included: convict surgeonfish (manini; *Acanthurus triostegus*) (0.16 per 50 m^2^ long-term average compared to 5.44 during lockdown); manybar goatfish (āti'ati'a; *Parupeneus multifasciatus*) (0.28 per 50 m^2^ long-term average compared to 2.33 during lockdown); blacktail snapper (to'au; *Lutjanus fulvus*) (0.06 per 50 m^2^ long-term average compared to 0.22 during lockdown); striated surgeonfish (maito; *Ctenochaetus striatus*) (9.61 per 50 m^2^ long-term average compared to 34.44 during lockdown); and daisy parrotfish (Pa'ati; *Chlorurus sordidus*) (7.98 per 50 m^2^ long-term average compared to 25.89 during lockdown) (electronic supplementary material, table S3). Four species were recorded for the first time during the lockdown (scarlet soldierfish [ī'ihi; *Myripristis pralinia*] [2.78 per 50 m^2^], whitesaddle goatfish (ahuru pa'a; *Parupeneus ciliatus*] [0.22 per 50 m^2^], Tahitian squirrelfish [māuna'una; *Sargocentron tiere*] [0.11 per 50 m^2^], and little spinefoot [pā'aua ra; *Siganus spinus*] [0.11 per 50 m^2^]) (electronic supplementary material, table S3).

Similar patterns were recorded among the non-harvested species within the nMPA barrier reef sites. The five non-harvested species that experienced the largest increases included: blackspot sergeant (pa'e'e; *Abudefduf sordidus*) (0.01 per 50 m^2^ long-term average compared to 0.56 during lockdown); tripletail wrasse (parahirahi; *Cheilinus trilobatus*) (0.01 per 50 m^2^ long-term average compared to 0.04 during lockdown); longnose butterflyfish (paraha utu roa; *Forcipiger longirostris*) (0.01 per 50 m^2^ long-term average compared to 0.22 during lockdown); dot dash butterflyfish (pāraharaha; *Chaetodon pelewensis*) (0.01 per 50 m^2^ long-term average compared to 0.11 during lockdown); and sailfin tang ('iriā'eo; *Zebrasoma velifer*) (0.01 per 50 m^2^ long-term average compared to 0.11 during lockdown) (electronic supplementary material, table S4). Four species were also recorded for the first time during the lockdown (spot-fin porcupinefish [tōtara; *Diodon hystrix*] [0.11 per 50 m^2^], whitetip reef shark [mamaru; *Triaenodon obesus*] [0.11 per 50 m^2^], whitespotted boxfish [moemoe; *Ostracion meleagris*] [0.22 per 50 m^2^], and melon butterflyfish [pāraharaha; *Chaetodon trifasciatus*] [2.11 per 50 m^2^]) (electronic supplementary material, table S4).

### 3.2. Fisher attitude and COVID-19

The majority of fishers (91%, 63 of 69) indicated that they were afraid of catching COVID-19. Additionally, most (74%, 51 of 69) stated that they, or at least one member of their family, was financially impacted by the crisis. Most (80%, 55 of 69) indicated that the lockdown led to them and their families changing their diet, turning to a heavier reliance on locally harvested fruits and vegetables, as well as fishes from nearby reefs, rather than relying on food purchased from stores. Of the six professional fishers that we interviewed, four said that they continued to fish as usual during the lockdown period, while the other two said that their businesses primarily relied on selling to local restaurants and hotels and, as these businesses were closed, they did not fish. Of the amateur fishers, most (71%, 45 of 63) respected the lockdown, with most stating that the risk of punishment (US$150 fine for first breach of lockdown, rising to US$2800 for each re-offence) was not worth the potential reward. Most of those that did breach the lockdown (83%, 15 of 18) said that they fished from the coast, rather than from a boat, and fished near dawn or dusk (89%, 16 of 18) to minimize their likelihood of being caught by the authorities. While the majority of amateur fishers respected MPA no-catch restrictions, four (6% of 63) stated that they fished within MPA boundaries. Interestingly, all of these people lived directly in front of an MPA and stated that they did this as they required fish for subsistence.

## 4. Discussion

The COVID-19 lockdown highlights the chronic effects that anthropogenic activity has on coral reef fish communities, and that the behaviour of animals in human-impacted environments can rapidly change in our absence. During the six-week lockdown period, no tourists were allowed to the island and residents were under a strict lockdown regime. This led to a sudden halt in recreational use of the inner barrier reef locations, which are used for recreational activities. We found that this reduction in recreational use was associated with increases in fish density during the lockdown period. This contrasted with the outer barrier reef sites, which did not experience changes in fish density during lockdown. Interviews with local fishers indicated that the fear of catching COVID-19 and being issued heavy fines led to most amateur fishers respecting the lockdown. Professional fishers kept fishing unless they were dependent on selling their catches to the businesses that rely on tourists (e.g. hotels and restaurants), in which case they too ceased activity. Overall, our results indicate that the sudden removal of human activity has positive effects on the behaviour of fish populations, which resembles the results of comparable studies on pollution levels [28–30], terrestrial species [31,32] and in other coral reef ecosystems [6,15,16].

Similar to other reports of short-lived environmental relief resulting from lockdowns [2,15], we found that the significant increase in the number of fish detected within the barrier reef sites during lockdown (i.e. areas commonly used for human recreation) subsequently returned to pre-lockdown levels following easing of lockdown regulations. Interestingly, our observed increase in fish density within the barrier reef sites did not extend to their outer slope sites, and our results remained consistent regardless of MPA status. This suggests that the effects of the lockdown did not have a uniform effect on coral reef fish communities, which may reflect the baseline level of human activity occurring at inner versus outer reef sites. For instance, given their distance from shore, outer reef slope sites are likely subject to less human activity than inshore locations, which provide a recreation/fishing space for a wider array of local residents and tourists (e.g. [33]). Subsequently, the difference in activity and associated disturbance to fish communities during the lockdown were likely less, resulting in the minimal change in the density observed [34–36]. As our surveys were conducted on adult fish, these patterns may be explained by either fish rapidly recolonizing these areas, or resident cryptic species becoming more conspicuous (i.e. venturing further from crevices) in the absence of human activity. Considering that we recorded increases in a broad spread of species, spanning those that are typically site attached (e.g. damselfishes and hawkfishes) to those that actively roam (e.g. surgeonfishes and parrotfishes), our data are consistent with both possibilities, suggesting that the abrupt removal of human activity has general benefits for coral reef fish communities, which is consistent with comparable terrestrial [37] and marine [6,15,16] research.

## 5. Conclusion

CRIOBE has been conducting long-term monitoring of the coral reefs around Moorea since 2004, and the COVID-19 lockdown provided an opportunity to rapidly quantify the influence of human presence on animal behaviour [8]. Our results provide evidence that the sudden decline in human activity was correlated with an increase in the density of fishes within the barrier reef sites, and no significant differences were observed on the outer slope sites. This suggests that human activities have a variable, but generally negative, influence on the behaviour of both harvested and non-harvested fish species and that these communities are highly responsive to reductions in human activity, at least where fishing is prohibited. Examination of these effects would not have been possible without CRIOBE's ongoing monitoring efforts, and as such this study showcases the value of long-term monitoring data, in particular for examining acute events. While most fishers limited their activity during the lockdown, fishing inside and outside of the MPA network continued, which reflected the greater reliance on harvesting seafood, as stores and other commercial supply lines became less reliable. Overall, this study demonstrates the effect that our presence has on the ecological communities that we interact with and shows the speed with which these communities can rapidly change when we are removed.

## Data Availability

An R Markdown file containing all analyses contained within this paper is published in the electronic supplementary material. The data are provided in electronic supplementary material [38].
